# Model development including interactions with multiple imputed data

**DOI:** 10.1186/1471-2288-14-136

**Published:** 2014-12-19

**Authors:** Gillian M Hendry, Rajen N Naidoo, Temesgen Zewotir, Delia North, Graciela Mentz

**Affiliations:** School of Mathematics, Statistics and Computer Science, University of KwaZulu-Natal, Westville Campus, University Road, Westville, Durban, South Africa; Discipline of Occupational and Environmental Health, School of Nursing and Public Health, University of KwaZulu-Natal, Durban, South Africa; Department of Environmental Health Sciences, School of Public Health, University of Michigan, 6655 SPH I, Ann Arbor, MI 48109-2029 USA

**Keywords:** Interactions, Missing data, Model development, Multiple imputation, Ordinal regression

## Abstract

**Background:**

Multiple imputation is a reliable tool to deal with missing data and is becoming increasingly popular in biostatistics. However, building a model with interactions that are not specified *a priori*, in the presence of missing data, presents a challenge. On the one hand, the interactions are needed to impute the data, while on the other hand, the data is needed to identify the interactions. The objective of this study was to present a way in which this challenge can be addressed.

**Methods:**

This paper investigates two strategies in which model development with interactions is achieved using a single data set generated from the Expectation Maximization (EM) algorithm. Imputation using both the fully conditional specification approach and the multivariate normal approach is carried out and results are compared. The strategies are illustrated with data from a study of ambient pollution and childhood asthma in Durban, South Africa.

**Results:**

The different approaches to model building and imputation yielded similar results despite the data being mainly categorical. Both strategies investigated for building the model using the multivariate normal imputed data resulted in the identical set of variables and interactions being identified; while models built using data imputed by fully conditional specification were marginally different for the two strategies. It was found that, for both imputation approaches, model building with backward elimination applied to the initial EM data set was easier to implement, and produced good results, compared to those from a complete case analysis.

**Conclusions:**

Developing a predictive model including interactions with data that suffers from missingness is easily done by identifying significant interactions and then applying backward elimination to a single data set imputed from the EM algorithm. It is hoped that this idea can be further developed and, by addressing this practical dilemma, there will be increased adoption of multiple imputation in medical research when data suffers from missingness.

## Background

It is not unusual to encounter missing data in epidemiological studies [1, 2]. Its presence affects the analysis of the data, and the methods employed in handling missing data can affect the results of the analysis. This could compromise conclusions drawn from the results. Types of missingness have been well documented [3]. Popular classifications are “missing completely at random” (MCAR – the missing values are independent of both observed and unobserved data); “missing at random” (MAR – the missing values are independent of unobserved data but may depend on observed data) and “not missing at random” (MNAR – the missing data depends on both observed and unobserved data).

Commonly, missing data is managed by simply dropping all cases that are not fully measured. However, such a complete case analysis can introduce bias into the results and, in some cases, wrong conclusions can be drawn [4]. While this approach is acceptable when the incomplete cases do not exceed 5% [5] and for which the missingness can be classified as MCAR, when these conditions are not met, alternative means of dealing with the missing data need to be considered. One such method that is increasingly being used is multiple imputation (MI) [6].

Imputation of missing data on a variable involves replacing the missing value by a value drawn from an estimate of the distribution of the variable [7]. Multiple imputation does not replace the missing item with a single predicted value, but rather imputes multiple values for each missing data item. These multiple imputations and the addition of random error to each imputed item ensures that the variation in the imputed values follows closer the true distribution of the original measure. Multiple imputation is successfully applied to data that is MAR and yields unbiased results with accurate estimates for the standard errors [7]. Unfortunately, the missingness mechanism is not usually fully known and is often a combination of more than one mechanism. However, by ensuring that the imputation model is more general than the analysis model, multiple imputation will usually produce sound results [8–11]. This is achieved by including, in the imputation model, variables that are related to the incomplete variables as well as those related to their missingness; the outcome variable; and all interactions that will be examined in the analysis.

Rubin [12] suggests that the need to include all possibly relevant predictors in the imputation model is demanding in practice. If interactions are selected *a priori*, it is a straightforward exercise to include them in the imputation model [9]. If, on the other hand, the relevant interactions have not been identified beforehand, then ideally all possible interactions should be included in the imputation model. This is neither practical nor, in some cases, possible [13, 14], particularly when the number of variables is large. While model development with multiple imputation has been documented [13, 15–17], none of these studies addresses the issue of how to include, in the imputation model, interactions that are not known *a priori*. Developing a model with many variables, in the presence of missing data, when predictor variables include not only main effects but also interactions that are not pre-selected, presents a challenge, and not extensively reported in the literature. On the one hand, the data is needed to identify relevant interactions; on the other hand, the interactions are needed to impute the data. This paper addresses this dilemma and suggests a method in which model development, including interactions, and analysis can be carried out when missing data is imputed using multiple imputation.

We propose to identify the relevant interactions using a single complete set of data generated using the expectation-maximization (EM) algorithm for covariance matrices and then include these interactions in the imputation model.

## Methods

### The data

The relationship between environmental, socio-economic and genetic factors and the respiratory health of children in the Durban South region of KwaZulu-Natal, South Africa using cross-sectional data was investigated. The data comes from research commissioned by the eThekwini Municipality, Durban, South Africa in 2004 to investigate possible causal effects of environmental and lifestyle factors on respiratory health in children [18]. Ethical approval was obtained from the Ethics Committee of the University of KwaZulu-Natal (Ref No.: E117/03). All the legal guardians of the child participants in this study gave written informed consent, participated voluntarily, and had the right to withdraw at any stage.

After an asthma symptoms screening survey, a sample of 423 primary school children were invited to participate in the study and from each participant multiple questionnaires were required to be completed. Of the 423 children included in the study, 382 that were deemed to have reliable data as well as complete data on the outcome variable, asthma severity, were used for this analysis. The removal of these children did not result in any selection bias.

Most of the predictor variables suffered from missing data. A study on the missingness mechanism was made prior to imputing the missing values. For each incomplete variable, an indicator variable was created and Chi-square analyses were performed to test whether either the incomplete variable or its missingness was related to observed values of other variables.

### Selection of interactions for the imputation model

In order to ensure that the imputation model is at least as complex as the analysis model, and that the assumption of MAR is plausible, it is necessary to include the outcome variable and all possible likely predictors for the analysis model, in the imputation model. The selection of the interaction terms presents difficulties [16, 17]. Comparable to the suggestion made by White et al [16], we have generated a single complete set of data using the EM algorithm for covariance matrices. The EM algorithm is an iterative procedure that can be used to create a complete data set in which all missing values are replaced by maximum likelihood (ML) values that are asymptotically unbiased. The process starts by replacing each missing value with an estimate calculated from a regression equation in which all the other variables are predictors. Once all the missing values have been replaced, a variance covariance matrix and a vector of means from the completed data are calculated. New regression equations are then formed to predict a new set of estimates for the missing values. This process is repeated until the variances, covariances and means converge, thus producing ML estimates of the parameters.

The complete data set generated from this process is then used for model development and the identification of interactions. In our application, convergence was achieved in 36 iterations.

### Multiple imputation

The imputation of multiple data sets was carried out using two different algorithms – multivariate normal imputation (MVNI) and fully conditional specification (FCS).

MVNI – This imputation algorithm, adopted by the NORM software [19], assumes the complete data (observed and missing values) follows a multivariate normal distribution. NORM uses a data augmentation (DA) procedure to impute multiple sets of data.

This two-step process makes use of the ML estimates from EM as parameter starting values. In the first step, DA randomly imputes the missing data using the assumed values of the parameters. In the second step, new parameter estimates are drawn from a Bayesian posterior distribution based on the observed and imputed data. The repetition of these two steps results in a Markov chain. DA converges when the distribution of parameter estimates stabilizes. Research has shown that DA nearly always converges in fewer cycles than does EM [8]. This enables one to estimate the cycle length, k, of DA as being any number at least as large as the number of iterations needed for EM to converge.

In order to impute m sets of data, DA is run for N = mk iterations and the data set at the end of every k^th^ cycle is saved.

Because the data contained categorical variables, some adjustments were necessary both before and after imputation. Before imputation, dummy coding was applied to all the categorical variables and interaction product terms with more than two categories. After imputation, sensible rounding [20] was used on these variables to prepare the data for analysis.

FCS – FCS, also termed “chained equations”, is the multiple imputation algorithm adopted by SPSS [21]. This is a more flexible approach to imputation in that it is designed to handle different types of variables (continuous, binary, categorical, ordinal) and does not assume multivariate normality of the data [6].

In practice, FCS involves running a series of regression models such that each variable with missing data is regressed on the other variables in the data set according to its distribution. So, for example, categorical variables will be modelled using logistic regression and continuous variables will be modelled using linear regression.

Imputation by FCS, as applied in SPSS, is also an iterative process that starts by imputing every missing value with random draws from the distribution of the non-missing values. Continuous variables are replaced with draws from a normal distribution and categorical variables are replaced with draws from a multinomial distribution. Azur et al [22] refer to these replacements as “place holders”.

Each iteration involves the following steps:

- Set the “place holders” of one variable that suffers from missing values back to missing
- Set up a regression equation, according to the distribution of the variable, with the observed values as the dependent variable and the other variables as independent variables
- Replace the missing values from this variable with predictions from the regression equation
- Repeat these steps for each variable that has missing values.

This forms one iteration of the process. At each iteration the imputed values are updated. This process is repeated for a specified number of iterations, n, after which the data set is retained as one complete imputed data set. The number of iterations, n, chosen so that the parameters from the regression models have stabilized, is generally about ten [23]. This entire process is repeated until the required number, m, of imputed data sets is generated.

Each of the m data sets were analysed with ordinal regression – the chosen method of analysis – and the results were combined using Rubin’s rules [4]. Although, in the past, it was widely thought that as few as 3 imputed data sets are needed to obtain good results and inferences, new studies have shown that this may, in fact, not be enough [24]. Studies have shown that there could be an important reduction in statistical power if m is small [9]. Graham et al [24] completed a simulation study on the number of imputations needed to attain maximum power. Their recommendations for the number of imputations, m, as a function of the fraction of missing information are summarized in Table 1. On the basis of the percentage of data missing in this study (5.3%), 20 sets of data were imputed.

**Table 1:** **Recommended number of imputations needed for varying fractions of missing data (Graham** [9]**)**

Fraction of missing data	0.1	0.3	0.5	0.7	0.9
Number of imputations	20	20	40	100	>100

### Model development

In order to develop the best model given the large number of variables available, the following three-stage process was followed. Firstly, all variables were purposefully selected as main effects. Secondly, in developing the full model, interactions were chosen one at a time in a stepwise manner such that the interaction that made the biggest significant improvement to the fit was added to the model. For this process a cut-off *p*-value of 0.05 was used. Thirdly, when no further improvement to the fit was possible, backward elimination was carried out to find the smallest model that was as good as the full model. Here a *p*-value of 0.10 was used for the stopping criterion.

### Model development with multiple imputation

In the setting of the multiple imputation process, we suggested two possible strategies that can be applied to carry out the model development process.

#### Strategy 1

All three stages of the model development process - the selection of main effects, identification of interactions as well as the backward elimination - are performed on the initial data set generated by the EM parameters. The variables and interactions identified by this process are incorporated into the imputation model. Interactions are treated differently, depending on which imputation method is used.

For MVNI as implemented in the NORM software, interactions with p categories are treated as categorical variables and coded into p-1 dummy variables before being added to the raw incomplete data. By way of an example: an interaction between gender (male/female) and smoking (yes/no) is broken down into separate categories – male/yes, male/no, female/yes and female/no – and binary coding (present/absent) is applied to the first three categories.

For FCS, the interaction is coded according to the possible categories. So, in the example above, male/yes = 1, male/no = 2, female/yes = 3 and female/no = 4.

The interactions as coded in the two scenarios above are merely treated as additional variables. This has been referred to as the ‘transform-then-impute’ method of dealing with interactions and, in a regression model that includes interactions, has been shown to yield good regression estimates, even though the imputed values are inconsistent with one another. In contrast to this is the ‘impute-then-transform’ method, also known as passive imputation, which yields plausible-looking imputed values but biased regression estimates [25].

This imputation model is then used to produce the m sets of imputed data. These are analysed individually and the results are combined using Rubin’s rules [4].

#### Strategy 2

Using the initial EM generated data set, the first two stages of the model development process are completed - selection of main effects and identification of interactions. These are then incorporated into the imputation model as before and m sets of imputed data are produced. Analysis, followed by the third stage of model development (backward elimination), is then applied to each of these data sets. The final selection of variables for the model includes those that are selected in at least 50% of the individual data sets. In the event that no variables satisfy the selection criterion, the condition can be relaxed to a lower percentage. Once these variables are established, analysis is carried out on each data set and the results are combined.

### Analysis

Analyses were carried out using the Statistical Package for Social Sciences (SPSS v17). Given that the outcome variable, asthma severity, is an ordinal measure, the chosen method of analysis for this data was ordinal regression. The three categories of the outcome variable are ‘none/mild intermittent asthma’; ‘mild persistent asthma’ and ‘moderate/severe asthma’. For all the analyses, logit was the chosen link function.

In addition to the analysis of the imputed data, a complete case analysis was carried out for comparative purposes. All main effects and interactions that were defined in stages 1 and 2 of the model building process were used with the complete case analysis and then backward elimination was applied to reduce the model.

## Results

### Data review

A total of 22 variables make up the data for this analysis. (1 interval and 21 categorical environmental, genetic and socio-economic variables) (Table 2). Of these variables, 18 (81.8%) experienced some missing data; a total of 166 (43.5%) of the subjects had incomplete data; and, overall, 445 (5.3%) items of data were missing. Missingness in variables ranged from 19.4% to less than 5%. Completely measured variables include age, gender, area and the outcome variable, asthma severity. The missing values follow a nonmonotonic pattern. The majority of non-response was as a result of whole sections or pages of questionnaires being left out. In some instances, one or more of the four questionnaires were missing. There were also numerous cases of seemingly random omissions of individual data items and, in some cases, it is evident that the required information was not known.

**Table 2:** **Variables, categories and the percentage missing**

Variable	Response category	% missing
Gender	male/female	0
Neonatal care	yes/no	3.7
Birth weight	up to 2.5 kg/>2.5 kg/don’t know	1.0
Fear in neighbourhood	yes/no	6.5
Smoked while pregnant	yes/no	50.
Smokers in the home	yes/no	0.3
Smoke exposure in vehicles	yes/no	7.6
Exercise	Up to once a week/2-4 times a week/>4 times a week	6.3
TV watching	Up to an hour a day/1-3 hours a day/>3 hours a day	6.5
Number people in home	1-4/5-7/8+	9.2
Income (monthly)	up to R1000/R1001-R4500/R4501-R10000/R10001+	19.4
Food availability	not always enough/enough	8.4
Perceived weight	overweight/underweight/correct weight	6.8
Work and wear	yes/no	3.7
Pets at home ever	yes/no	1.0
Area	South Durban/North Durban	0
Breakfast habits	Not every day/daily	6.5
Violence experienced	yes/no	7.3
Attacked with weapons	yes/no	7.3
Stove type	paraffin/gas/electric/none	9.9
Age		0
Asthma severity	Moderate-severe/mild persistent/mild intermittent/no asthma	0

Results from the chi-square analysis, to test whether either the incomplete variable or its missingness was related to observed values of other variables, showed that for all but three of the incomplete variables, missingness was associated with measured values in other variables; and all variables were associated with at least one other variable in the set. Thus missingness for these variables can be assumed to be MAR. However, it cannot be ruled out that there exists some MNAR mechanism in the data. Further analysis showed that the distribution of the outcome variable, asthma severity, is the same (in a statistical sense) for whether data is present or missing for all variables except ‘food availability’, where fewer than expected of those with missing data on the food variable did not have asthma. Because asthma severity is related to the missingness of ‘food availability’ but not to ‘food availability’ itself, the inclusion of asthma severity in the imputation model will make the MAR assumption for ‘food availability’ more plausible [9].

### Model development

#### Imputed data -MVNI

The two different strategies suggested for building the model using the imputed data resulted in the identical set of variables and interactions being identified. In each case 17 main effects and 10 interactions were included in the final model (Table 3). While fewer than half of the main effects were significant, the interactions in which these variables were involved were largely significant. Main effects dropped from the model include birth weight, perceived weight, weapons and stove type. However, these were left in the imputation model as they were shown to be associated with other variables and/or their missingness.

**Table 3:** **Estimated coefficients (EST) and standard errors (SE) for the predictors selected in the different analyses**

Predictor	Reference	Category	CC (N = 216)		MVNI (N = 382)		FCS1 (N = 382)		FCS2 (N = 382)	
	Category		EST	SE	EST	SE	EST	SE	EST	SE
Gender	Female	Male	-0.441	0.674	0.129	0.398	0.030	0.391	0.017	0.390
Neonatal care	No	Yes	2.484*	0.723	1.103*	0.444	1.112*	0.450	1.085*	0.446
Fear	No	Yes	-1.169	0.649	-0.958*	0.431	-1.009*	0.451	-1.073*	0.444
Smoked while pregnant	No	Yes	4.256*	1.237	1.019	0.736	0.885	0.693	0	
Smokers in home	No	Yes	0.939	0.537	0.742*	0.352	0.761*	0.341	0.801*	0.335
Smoke in vehicles	No	Yes	-2.584*	0.921	-0.253	1.068	-0.308	1.011	-0.323	1.015
Exercise	>4 times a week	Up to once a week	2.805*	1.227	0.892	0.761	0.692	0.756	0.624	0.731
		2 – 4 times a week	3.313*	1.229	1.039	0.717	0.936	0.718	0.738	0.680
TV watching	>3 hours a day	Up to 1 hour a day	-0.566	0.854	0.399	0.684	0.327	0.669	0.346	0.657
		1 – 3 hours a day	0.304	0.769	0.641	0.639	0.525	0.630	0.569	0.618
Number people in home	8+	1 - 4	0		1.084	0.554	1.060*	0.539	1.101*	0.526
		5 - 7	0		0.226	0.552	0.254	0.551	0.250	0.540
Income	R100001+	up to R1000	2.840*	1.257	0.695	0.8	0.787	0.789	0.823	0.778
		R1001 – R4500	1.285	1.203	0.209	0.797	0.489	0.754	0.431	0.754
		R4501 – R10000	1.933	1.17	1.428	0.783	1.401*	0.692	1.356	0.692
Food availability	Enough	Not always enough	-0.575	0.64	0.604	0.503	0.665	0.464	0.677	0.455
Perceived weight	Correct weight	Overweight	-0.230	0.743	0		0		0	
		Underweight	2.369*	0.97	0		0		0	
Work’nWear	No	Yes	0		-0.635	0.626	-0.543	0.629	-0.478	0.622
Pets ever	No	Yes	-3.770*	0.994	1.658*	0.501	-1.483*	0.503	-1.413*	0.467
Area	North Durban	South Durban	6.278*	1.461	2.042*	0.76	1.948*	0.737	1.597*	0.671
Breakfast habits	Daily	Not daily	-4.098	3.04	-0.492	1.512	-0.234	1.548	-0.110	1.518
Violence	No	Yes	0		-0.817*	0.382	-0.741*	0.377	-0.715	0.373
Weapons	No	Yes	-1.147*	0.555	0		0		0	
Age			-1.068*	0.438	-0.79*	0.254	-0.833*	0.268	-0.834*	0.265
**Predictor**	**Reference**	**Category**	**CC (N = 216)**		**MVNI (N = 382)**		**FCS1 (N = 382)**		**FCS2 (N = 382)**	
	**Category**		**EST**	**SE**	**EST**	**SE**	**EST**	**SE**	**EST**	**SE**
Fear*Breakfast	No/daily	Yes/not daily	2.635*	1.219	2.047*	0.866	2.123*	0.916	2.185*	0.911
Gender*SmokeVehicle	Female/No	Male/yes	5.092*	1.342	2.535*	1.034	2.431*	0.977	2.464*	0.971
SmokeVehicle*TV	No/>3 hrs	Yes/up to 1 hr	0		0.891	1.298	0.675	1.265	0.722	1.250
		Yes/1 – 3 hrs	0		-2.184*	1.085	-1.975	1.034	-2.002	1.037
Food*Age	enough/	Not always enough/	1.762*	0.743	0.925*	0.396	0.786*	0.385	0.778*	0.364
Exercise*Area	>4 times/ND	< once a week/SD	-4.573*	1.533	-1.41	1.031	-1.255	0.954	-1.125	0.923
		2 – 4 times/SD	-6.331*	1.627	-1.981*	0.913	-1.805*	0.896	-1.551	0.850
Income*Breakfast	> R10000/daily	≤R1000/not daily	-4.051	2.5	-3.921*	1.8	-3.666*	1.731	-3.808*	1.733
		R1001-R4500/not daily	0.414	2.408	-1.218	1.636	-1.439	1.530	-1.513	1.516
		R4501-R10000/not daily	2.479	2.395	-1.374	1.541	-1.568	1.454	-1.715	1.431
TV*Breakfast	>3 hrs/daily	≤1 hr/not daily	6.310*	2.213	2.573*	1.259	2.051	1.192	1.976	1.186
		1-3 hrs/not daily	1.974	2.154	0.192	1.109	0.270	1.112	0.192	1.103
SmokeVehicle*Age	no/	yes/	0		0.814*	0.375	0.809*	0.348	0.782*	0.341
Smoke preg*Area	no/ND	yes/SD	-5.118*	2.101	-1.875	1.363	-1.663	1.291	0	
Work’nWear*Breakfast	no/not daily	yes/daily	0		2.349*	1.076	2.095	1.070	2.165*	1.090

ND – North Durban; SD – South Durban; preg – pregnant.

CC – Complete case.

MVNI – Multiple imputed MVNI strategies 1 and 2.

FCS1 -Multiple imputed FCS strategy 1.

FCS2 -Multiple imputed FCS strategy 2.

*Significant at the 0.05 level.

#### Imputed data -FCS

Model development following strategy 1 resulted in the identical model as identified when applying MVNI imputation. The set of significant variables from the two analyses were, however, not the same. Two main effects and three interactions differed in their significance. With strategy 2, the variable ‘Smoke while pregnant’ and its interaction with ‘area’ did not make the cut to be included in the model. These two variables were significant in only 9 of the 20 individual analyses, whereas, they were significant in 10 of the 20 analyses when MVNI imputation was applied.

#### Complete case analysis

The complete case analysis was based on 216 complete cases, representing 56.5% of the total available cases. The final model contained 16 main effects and 7 interactions (Table 3).

The main effects selected with the complete case data compared to those selected with the imputed data differed slightly. ‘Perceived weight’ and ‘weapons’ are the only variables that are in the complete case model but not in the imputed data model. Three of the 10 interactions and three of the main effects from the imputed data models were not retained in the complete case model. The models from the imputed data contained more variables than the complete case model.

### Analysis

Results of the three different analyses of the imputed data (Table 3) are, in general, very similar. The size and direction of association between asthma severity and all the predictor variables, as well as the standard errors (SE’s) of the estimated coefficients are consistent across both types of imputation as well as for both model building strategies. Even though some differences in the significance of certain predictors did occur, in all cases the p-values showing significance of these predictors were only marginally different from the 5% cut-off value.

A comparison of results of the complete case analysis(CC) with the other analyses shows that the standard errors of the estimated coefficients for the CC analysis are appreciably larger in all but the one predictor variable – ‘smoke in vehicle’. There are also noticeable differences in the magnitude of the estimated coefficients for the CC analysis as compared to the other analyses. Contradictions are also present regarding the relationship with asthma severity for some of the predictors.

### Diagnostics

**Figure 1:**
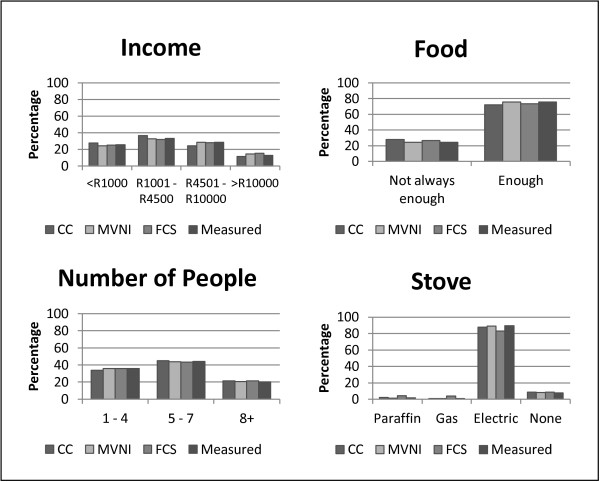
**Differences in measured (observed) and imputed data.** A comparison of the distributions of the 4 variables with the most missing data for the complete case data (CC), MVNI imputed data, FCS imputed data and measured data.

In order to confirm that the imputed values are reasonable, each variable with missing data in excess of 8% was examined to identify variables with large differences between the measured and imputed. The variables considered included income, stove type, number of people and food availability (Figure 1). The Kolmogorov-Smirnov test was applied to assess whether significant differences exist between the distributions of the imputed data – both MVNI imputed and FCS imputed – and the measured data [26]. No significant differences were found.

In analysis testing for significant differences between the distributions of the imputed data sets and the complete case data, no significant differences were found.

Another useful diagnostic that gives an indication of the stability of the estimates resulting from multiple imputation is the degrees of freedom (df) associated with the t-value in Rubin’s rules and adapted from Schafer [8, 9]. The df associated with multiple imputation is not the same as the df found in other statistical concepts and rather is a ‘measure’ of the ratio of the within-imputation variance to the between-imputation variance. In this study, df ranged from 130.54 to 9073.51 for the NORM imputations and from 138.88 to 15135.431 for the FCS imputations which, being large compared to the number of imputed sets, is an indication that the estimates have stabilized and can be trusted.

## Discussion

In this study investigating methods for addressing missing data, specifically when including interactions in the analysis, we found support for building the model using an EM generated set of data and then applying multiple imputation as a robust method to address this common shortcoming in epidemiological studies.

Epidemiological studies frequently suffer from missing data. Many researchers avoid this problem by dropping all cases with data missing on any variable and carrying out what is known as a complete case analysis. An advantage of this type of analysis is that it is computationally easy to apply and can be done with any reputable commercial software package. However, unless the data is MCAR, the values of the estimated coefficients produced with this analysis may be biased. Moreover, when the missingness is not only a function of the covariate(s) but also of the outcome variable, then the bias from a complete case analysis is heightened [27]. Although complete case analysis and other *ad hoc* methods, like mean substitution and the missing-indicator method, are still widely used, researchers are becoming more aware of the perils of applying such methods and many are now employing multiple imputation methods to address the missingness in their data. While results from multiple imputation will be unbiased when data is MAR, it has been suggested that even when it is MNAR, adequately dealing with as much of the missingness mechanism as possible will usually produce sound results [8–11]. This is achieved by including auxiliary variables – those variables related to the missingness but not necessarily included in the analysis, interactions and the outcome variable in the imputation model.

While much has been published on the application of multiple imputation to epidemiological studies, there is limited literature that deals with model building in the presence of missing data, and more specifically model building including interactions. The aim of this paper was to demonstrate a simple and easily applied strategy to build interactions, which are not known up front, into a model while at the same time imputing the missing data.

The dilemma that we faced was a practical one. It is possible for the interactions to be added after imputation. This is termed passive imputation or ‘impute-then-transform’. However, it has been shown that including interactions, as product terms, before imputation produces superior results than if the imputations are done first and the interactions are added at the analysis stage [25]. For the best results, the identified interactions should be included in the imputation model along with the predictor variables, the auxiliary variables and the outcome variable. However, how can the interactions be identified and the best model built, when the data is incomplete?

Two strategies for model building, S1 and S2, were explored – both utilizing a single imputed data set generated from the ML parameter estimates produced from the EM algorithm for covariance matrices.

Imputation was carried out with both multivariate normal imputation (MVNI) and the more flexible fully conditioned specification (FCS). The same set of 17 predictor variables and 10 interactions for the best model were identified when applying MVNI with both strategies S1 and S2, as well as with the application of FCS and strategy S1. FCS with strategy S2 failed to include one of these predictors and an associated interaction in its best model. Since these dropped variables did not alter the interpretation of the results, it would seem that both strategies for model building are equally effective. The advantage of S1 over S2 is that it is easier and less time-consuming to execute and therefore probably the preferred choice.

In comparison to the model variables selected from the imputed data, fewer variables were selected for the model on the complete case data. This is most likely caused by the enormous reduction in cases and the subsequent loss of power.

A total of 5.3% missing items spread across 81.8% of variables, affecting 43.5% of cases was present in the dataset used for this analysis. Examination of the missingness revealed that it is possible that the missingness mechanism present in this data is a combination of MCAR, MAR and MNAR. Analysis of the relationships between both the missingness of the variables and the variables themselves confirmed that significant relationships exist between each of the variables and at least one other variable in the set; furthermore, the missingness of all but three of the variables is significantly related to at least one other variable in the set.

For reliable and unbiased results to be obtained from a complete case analysis, the data is required to be MCAR, which is clearly not the case here. Furthermore, although this means of dealing with missing data is acceptable when the lost cases amount to no more that 5%, this data set is reduced by over 40% which will inevitably have a negative effect on the outcome of the analysis.

On the other hand, multiple imputation, if applied correctly, is able to produce sound results when the data is MAR and it has been shown that even when the data is MNAR, the effects of this mechanism are often surprisingly minimal [11]. In order to ensure that the imputation model was general enough to encompass the subsequent analysis, the outcome variable, interactions and variables related to either the incomplete variables or their missingness or both were included in the imputation model. By including variables that are correlated with each incomplete variable but not its missingness, we expect that the additional information will cause a decrease in the standard errors and hence an increase in efficiency and statistical power [10]. If there is an element of MNAR present in the data, the inclusion of these variables in the imputation model should lessen the bias and make the assumption of MAR more plausible.

It is unclear as to how many variables and interactions, given the sample size available, can be reliably assessed with multiple imputation applications. It seems that this depends to some extent on the software being used. In some cases, convergence of large models is a problem in that it can make the imputation process unacceptably slow [16]. Graham and Schafer [28], in a study using NORM to perform the imputations found that results were quite acceptable “even with sample sizes as low as 50, even with as much as 50% missing from most variables, and even with relatively large and complex models”. In a study on the imputation of categorical data [29] it was found that, while problems exist when imputing using a variant of NORM designed to deal with categorical data when many variables are present, the same limitations are not problematic for NORM. In another study [30] on the inclusion of continuous auxiliary variables in the imputation model, the authors suggest the ratio of cases with complete data to variables should be at least 3:1. Given these guidelines, we found that convergence for both imputation methods was achieved quickly and reliably. Furthermore, even with the dummy coding of all the categorical variables and the interactions, the ratio of complete cases to variables far exceeds 3:1. We are therefore confident that our results are reliable.

Diagnostic tests on the distributions of the imputed data showed that data imputed both with MVNI and FCS were not significantly different from either the measured data or the CC data. These results confirm findings that multiple imputation with MVNI incorporating sensible rounding should work in most situations [14], even in the presence of binary and ordinal variables [6].

The diagnostic measure, df, also indicated that the estimates obtained from both multiple imputation methods have stabilized and are therefore trustworthy.

Analysis of the two sets of imputed data yielded very similar results. This is consistent with findings from a study comparing the two imputation approaches [6] where it was found that “similar results can be expected from FCS and MVNI in a standard regression analysis involving variously scaled variables”. The magnitude of the standard errors and the magnitude and direction of the estimated coefficients were consistent across both these imputation types and for both model building strategies. While there were some inconsistencies in the significance of predictors, these did not affect the overall interpretation of the associations between asthma severity and the factors included on the models.

A comparison of results for the complete case analysis and the analyses of the imputed data showed that standard errors for the estimated coefficients from the analysis of the imputed data were, in all but one case, considerably smaller than those from the complete case analysis. These smaller standard errors resulted in greater accuracy of the estimated coefficients. This increased precision indicates the superior efficiency and statistical power obtained for the analysis of the imputed data. The inconsistencies in the signs of the estimates and the significance of the predictors could result from the non-random fashion in which cases are dropped for the complete case analysis which may distort the joint distribution among the variables. The resulting bias in point estimates could lead to misidentification of significant predictors [31]. Another important factor that would negatively affect results of the complete case analysis is that the missingness mechanism present in the data is not confined to being MCAR. While multiple imputation methods produce unbiased parameter estimates when the missingness is MAR, this is not the case with complete case analysis. This missingness mechanism factor could also have added to the large difference in magnitude of the standard errors for the complete case analysis as compared to the imputed data analysis that, some would argue, could not be explained on the basis of sample size alone.

These results are consistent with what we expect given the significant reduction in cases for the complete case analysis and the missingness mechanism present in the data that would almost certainly result in a loss of power and the introduction of bias into estimates.

Given the rigid processes followed in the imputation of the data and subsequent analyses, we would suggest that the results from the imputed data can be considered reliable. On the other hand, the results from the complete case analysis should be treated with caution.

## Conclusions

With the development of readily available and easily implemented software, multiple imputation methods for dealing with missing data are becoming more popular in epidemiological studies that have incomplete measured variables. A critical part of the imputation process is the inclusion of those variables that are correlated with missingness as well as the interactions to be used in the analysis process. While this can present a practical challenge if the interactions are not specified *a priori*, we have illustrated one possible approach that effectively identifies the best main effects and interactions for a model in the presence of missing data and at the same time, imputes the data items that are missing. Undoubtedly, further testing of these strategies on other data sets is needed. It is hoped that the ideas presented in this paper can be further explored and developed so that, by addressing this practical dilemma, more medical researchers will be able to apply multiple imputation when data suffers from missingness.

## Abbreviations

CC: Complete case
df: Degrees of freedom
EM: Expectation Maximization
EST: Estimates
FCS: Fully conditional specification
MAR: Missing at random
MCAR: Missing completely at random
MI: Multiple imputation
MNAR: Not missing at random
MVNI: Multivariate normal imputation
ND: North Durban
SD: South Durban
SE: Standard errors.
